# Difficulties and challenges experienced by nurses in eldercare institutions in Albania: A qualitative content analysis

**DOI:** 10.1371/journal.pone.0300774

**Published:** 2024-03-27

**Authors:** Nertila Podgorica, Emiljano Pjetri, Andreas W. Müller (M. A.), Susanne Perkhofer

**Affiliations:** 1 Nursing Department, Health University of Applied Sciences Tyrol FH Gesundheit Tirol, Innsbruck, Austria; 2 Department of Nursing Science and Gerontology, UMIT - Private University for Health Sciences, Medical Informatics and Technology, Hall in Tyrol, Austria; 3 Nursing Department, University of Shkodra “Luigj Gurakuqi,” Shkoder, Albania; 4 Department of History, Martin-Luther-Universität Halle/Wittenberg, Wittenberg, Germany; 5 Research and Innovation Unit, Health University of Applied Sciences Tyrol/FH Gesundheit Tirol, Innsbruck, Austria; University of KwaZulu-Natal College of Health Sciences, SOUTH AFRICA

## Abstract

**Introduction:**

The global and Albanian populations of elderly people are steadily increasing. It is estimated that the number of elderly adults requiring care in Albania will rise from 90.9 thousand to 130.4 thousand by 2030. Despite the envisaged increase in the number and life expectancy of the elderly population in Albania, which will result in an increased demand for nursing care, little is known about the difficulties and challenges that nurses face while providing care for elderly Albanian individuals.

**Aim:**

To explore the difficulties and challenges nurses experience while caring for elderly people in Albanian eldercare institutions.

**Methods:**

The study employed a qualitative design using purposive sampling of 20 nurses in 8 eldercare institutions who participated in face-to-face semi-structured interviews. The audio-recorded interviews were transcribed and subsequently subjected to analysis using Graneheim and Lundman’s qualitative conventional content analysis. Data analysis was supported by the qualitative data analysis software MAXQDA 2020. The reporting of this study followed the consolidated criteria for reporting qualitative research (COREQ) checklist.

**Results:**

Five key categories emerged from data analysis: (1) professional difficulties, (2) educational difficulties, (3) relationship challenges, (4) increased mental stress, and (5) participation in advocacy. This study showed that nursing staff experienced many barriers, challenges, and unmet needs when implementing care for elderly people in long-term care facilities.

**Conclusion:**

The findings indicate that nurses working in eldercare institutions faced significant challenges in caring for elderly people. Nurses need more legal, financial, educational, and emotional support. The study indicates that more organizational and national support is necessary for nursing staff to care for elderly people in eldercare Albanian institutions properly. Eldercare institution leaders need to recognize the importance of their role in overcoming the barriers and providing adequate support for their staff in caring for elderly people.

## Introduction

Albania is currently experiencing a rapid and irreversible aging of its population. According to a report by the Albanian Institute of Statistics, by 2031, the population will slightly decrease to 2.74 million, while the old-age dependency ratio is forecast to reach 32.7%. Furthermore, the number of persons aged 65 or older is expected to increase to 21.8% of the total population. Statistical data from the United Nations report that the population in Albania is expected to decrease to 2.42 million by 2050 and 1.94 million by 2070, and the elderly dependency ratio will increase to 40.7% by 2050 and 76.1% by 2070 [1–3]. It is estimated that the number of older people in Albania potentially in need of long-term care will increase from 90.9 thousand to 130.4 thousand by 2030 [1, 3, 4]. These statistical data show that the healthcare system in Albania is facing challenges and that there are difficulties and problems in the long-term care (LTC) system, which is still underdeveloped in Albania [1, 5]. Albania currently lacks a formal LTC system [1, 3], as there is no official definition of LTC. There are provisions for long-term care in various laws, such as healthcare, social welfare, and social security, but a proper system is not defined [1, 6]. The Ministry of Health and Social Protection of Albania (MHSP) manages all these services. Elderly services are provided through social services in public service centers, such as community, residential, and regular or palliative day centers. These public services are financed from the state budget and municipal budgets; social services are provided in non-public service centers [5, 7–10]. Nonetheless, these public service centers face limitations in capacity and capability to provide adequate eldercare. They serve different groups of people, including people over 60 who are disabled or chronically ill or who have been abandoned by their families. There are 39 residential centers for the elderly in Albania, of which 14 provide services in the Tirana district. In comparison, the remaining 25 offer services in Korça (6 centers), Shkodra (5 centers), Berat (3 centers), Durres, Vlorë and Elbasan (2 centers each), Lezhë, Dibër, Gjirokastër, Fier and Kukës (1 center each) [1, 11]. The services for older people in these centers are guaranteed following the standards approved by the Albanian government: multidisciplinary teams assess the needs of older people and design and implement the time plan of individual interventions to meet the identified needs [4, 5, 10]. The staff of these centers is mainly composed of doctors and nurses. At the same time, there are only a few social workers and nursing assistants, even though the profession of social worker and nursing assistant is provided for in the authorized structure [3, 4]. This occurs because neither university curricula nor training institutions in Albania provide adequate training for nursing assistants and social workers in caring for older people [8, 9]. Nurses are considered the backbone of the healthcare system in providing essential healthcare services to the population. The knowledge, training, behavior, and care nurses provide significantly impact the quality of care, as studies indicate that care providers’ perspective influences the quality of care delivered to older individuals [12]. Nurses face various problems and difficulties when implementing nursing care and meeting their responsibilities to older people at eldercare institutions. These problems and challenges are related to the facilities’ physical environment and technical equipment, as well as the educational level of nurses [4, 8, 13]. The lack of staff training and a suitable environment is proven by the evidence to affect the quality of care provided to older people in eldercare facilities [9]. Empirical data revealed that nurses face difficulties in caring for the elderly due to communication and relationship issues with them, their relatives, or staff [8, 10, 14]. While working in LTC facilities, nurses care for people with dementia and other elderly with various health conditions who express uncertainty in decision-making regarding therapy or other decisions, especially at the end of their lives. As a result of being confronted with different moral dilemmas and being unable to protect older people, the nurses encounter various emotional problems [15, 16]. These challenges intensify when elderly residents exhibit increased aggressive behavior, leading to psychological distress in nurses, reduced working hours, attrition from the profession, and, ultimately, a shortage of nurses [14, 17]. In addition to the above issues, nurses working in LTC facilities confront many other difficulties and challenges in providing qualitative care to older people [14]. Since there is insufficient data on nurses’ challenges and difficulties while caring for older people in eldercare institutions in Albania, the evidence mentioned above comes mainly from studies conducted in other countries [1, 9, 14]. Most older individuals in Albania receive care from their families, particularly when they face serious illnesses or lose the ability to work [1, 3, 4, 6]. Older individuals in Albania have been cared for by family members, with children taking on the responsibility until the end of their parents’ lives [8]. However, this traditional care system is undergoing a rapid transformation [10] due to increased life expectancy, low fertility, increased female employment, the migration wave, and changes in Albanian culture. These changes have heightened the demand for care in eldercare institutions, as older individuals have fewer opportunities to reside with their adult children. The swift pace of these changes has resulted in a dearth of studies examining the experiences of nurses and the difficulties they encounter in caring for the elderly within Albanian eldercare institutions. Only a few studies have identified the barriers that affect the care of older people in Albania.

Hence, this study explored the difficulties and challenges nurses experience while caring for elderly people in Albanian eldercare institutions.

## Materials and methods

### Study design and setting

This qualitative study was conducted in 2019–2020 using the conventional content analysis approach. This method facilitates the depiction of experiences and the identification, summarization, and categorization of manifest and latent content within lived experiences [18]. Additionally, it aligns with the study’s objective, aiming to delve into the difficulties and challenges encountered by nurses providing care for elderly individuals in eldercare institutions in Albania.

Participants of this study were recruited from four different types of institutions, such as daily eldercare centers, public nursing homes, palliative care centers, and private nursing homes (Table 1), as there is no infrastructure for a long-term care system in Albania [1, 8].

**Table 1:** Frequency of data collection.

Working Institution	Institutions included (number)	Interviews (number)
**Public Nursing Home**	3	9
**Private Nursing Home**	2	4
**Palliatice Care Center**	2	4
Daily Eldercare Center	1	3
**Total**	**8**	**20**

### Study population and eligibility criteria

Nurses meeting the inclusion criteria were invited to participate in the research. The inclusion criteria comprised the nurses’ voluntary participation in the study, a minimum of one year of clinical work experience in providing care for older individuals in eldercare facilities, proficiency in Albanian communication, strong verbal skills, and the capability to articulate and share their experiences in detail. Before the researchers started interviewing, they tested the interview guide with two nurses to ensure the questions were clear, understandable, and easy to answer. These two nurses did not participate in the study, and their interviews were not included in the analysis.

### Sampling procedure and sample size

A purposive sample of nurses was recruited through social contact with nurses and directors of each facility based on their potential to provide information about their nursing knowledge and experiences for the study [19]. The initial 30 contacts who met the inclusion criteria and were willing to describe their nursing experiences consistent with the purpose of the study received a consent form by mail or e-mail and were verbally informed about the project. Once the participants signed and accepted, the consent form was mailed to the principal investigators before the start of the interview. Of these nurses, only 20 were interviewed, and the sample size was determined based on the principles of saturation as proposed by Malterud et al. [20]. Sampling was discontinued when no new information was obtained from study participants. All nurses voluntarily participated and were free to stop or withdraw from participating at any time without negative consequences.

### Data collection tool

A semi-structured interview guide was employed in this study, developed based on a review of the literature and modified to align with the specific objectives of the research [21]. The guide was used to collect detailed data regarding the difficulties and challenges nurses face when caring for the elderly living in eldercare institutions in Albania. An information sheet was used to manage the sociodemographic data of the study participants. The interview guide, initially prepared in English, underwent translation into Albanian (the national language) to ensure better understanding by the participants. To verify the appropriateness of the questions, a testing phase was conducted before the commencement of data collection.

### Data collection procedure

After obtaining participants’ informed consent, data was collected through in-depth and semi-structured individual face-to-face interviews (Table 2). The interviews, conducted by leading Albanian researchers (NP, EP) possessing a Ph.D. in nursing science and expertise in qualitative research, occurred between June 2019 and March 2020. Before the interviews, researchers communicated with participants, aiming to establish a friendly atmosphere. This involved introducing themselves, describing the study’s objectives and the reasons for their interest in the subject, and responding to possible questions from the participants. These efforts contributed to building a positive rapport and trust between researchers and participants. Additionally, participants received assurances regarding their freedom to answer questions and participate voluntarily, the confidentiality of their information, and the option to withdraw from the study at any point. Participants were given the autonomy to choose the location and timing of their interviews.

**Table 2:** Interview guide.

Main questions	
**1**	Could you tell me about your work in your eldercare institution?
	**Probe:** workplace, age, working experience in years, marital status, education, training.
**2**	What do you think about elderly/geriatric care?
	**Probe:** conditions in the institutions, preparation related to elderly adult care, guidance, and support received.
**3**	Could you tell me about your experiences caring for elderly residents in your institution?
	**Probe:** How do you prepare, perform procedures, communicate with residents, support the physical and psychological needs of residents, and address the physical and psychological problems that nurses face?
**4**	Could you tell me about your experiences related to other staff members’ experiences caring for older adult residents in your institution?
	**Probe:** physical, emotional, and psychological reactions of peers, co-workers, and family members.
**5**	Could you tell me what difficulties and challenges you experience when caring for an elderly adult resident?
	**Probe:** difficulties, barriers, and challenges while caring for elderly adults with physical or mental issues.
**6**	Is there anything else you would like to add related to providing care to elderly residents?
**7**	What was it like to participate in this interview?
	**Probe:** anything related to the questions, how the interview was organized, and any suggestions.

Interviews were conducted without time constraints, scheduled outside regular working hours, in a separate room at the participant’s workplace, ensuring privacy with only the interviewer and participant present. The interview process continued until all relevant experiences of the participants had been fully explained, and comprehensive responses had been obtained from the interviewees. A digital audio recorder was used to record the interviews. In semi-structured interviews, the questions are not fixed and predetermined but evolved based on the interview process. Follow-up questions followed each interview to obtain further information or clarify the participants’ answers. The interviews were digitally recorded in Albanian, and researchers noted non-verbal data such as mood, tone of voice, facial expressions, and posture during the sessions. Interviews lasted 40–70 minutes and continued until saturation occurred after the 15th interview, but after coding these interviews, all authors agreed to conduct more interviews to ensure saturation [20, 22]. The first 15 interviews were conducted between June and December 2019, two others in January 2020, and three additional interviews were conducted between February 27 and March 5, 2020, before the COVID-19 restrictions started in Albania. In all, 20 in-depth individual interviews with nurses were completed. There were no withdrawals from the study and no repetition of any interviews.

### Data analysis

The data underwent analysis following Graneheim and Lundman’s qualitative conventional content analysis [22]. Data analysis began shortly after the first interview by manually transcribing the recorded audio data and typing it directly into the computer using Microsoft Word software. After anonymizing the transcriptions by removing personal identifiers and assigning unique identification numbers, the transcripts were reviewed for accuracy using spot-checking, taking a small set of transcripts (3 of 20) by the researchers who speak Albanian and English (NP; EP), who read and reread all the transcripts carefully and translated them afterward into English following Santos et al. [23] strategy for translating qualitative interviews and research reports which suggest having language experts on research teams and translation and back-translation issues directly considered within the team. A professional translator then checked the translated transcripts. The back translation was done again by the same researchers. In the next step, three researchers (NP, EP, and AM) repeated the reading of the transcripts with an open mind to gain an overall impression and a general understanding of each transcript. Transcribed and translated data sets were imported into MAXQDA 2020, a software supporting qualitative data organization and coding [24]. In the next step, open data coding began by carefully reading the data line-by-line, extracting and coding each word, phrase, or paragraph aligned with the study’s purpose. In the categorization phase, codes were consistently compared and ranked according to their affiliations, including their differences and similarities. Codes were then subcategorized, sorted, and combined into fewer, more comprehensive categories. To assign findings to the same category, researchers approached the text with a degree of interpretation (i.e., open to underlying meanings). The first and second authors did the coding and initial categorization independently, and the third did the validation and suggestions for changes. This iterative and non-linear process [18, 25] moved between parts and the whole until no new codes or categories could be added. All subcategories and categories had been defined, and the final coding scheme was created by the first three authors, one of whom was a senior female researcher with a Ph.D. in nursing science and extensive experience in qualitative research, and the other two male authors, one of whom was a lecturer with a Ph.D. in nursing and the other an expert in qualitative research in social and medical sciences. The other authors then reviewed the coding scheme and agreed upon or discussed it until a consensus was reached.

**Fig 1:**
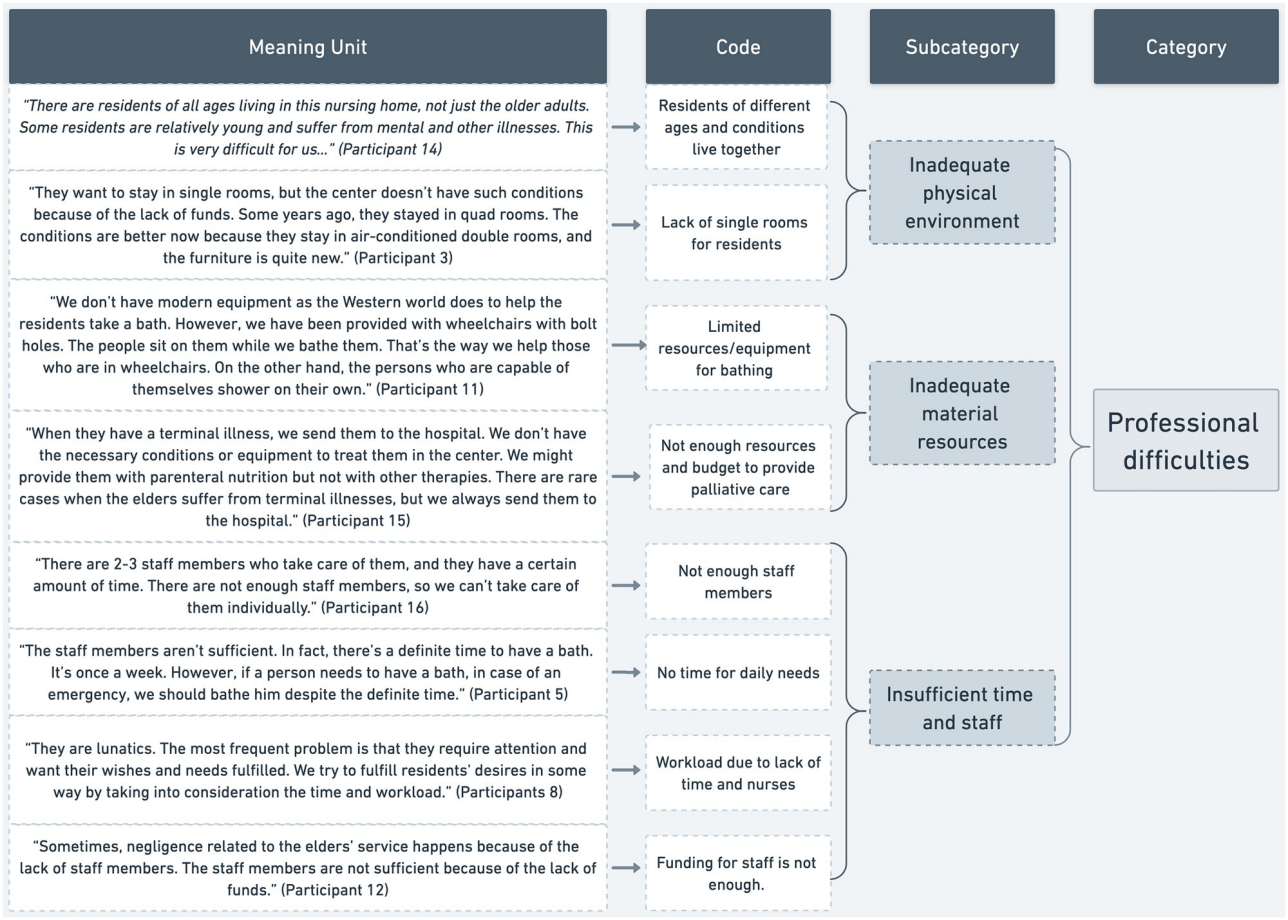
Examples of extracting codes, subcategories, and categories.

To ensure the reliability of the findings, the authors discussed the meanings, subcategories, and categories that emerged from the analysis following the specific objectives of this study. Additionally, meetings with study participants were conducted to ensure accuracy, facilitate discussion, and seek consensus on the identified subcategories and categories [26]. Participants’ suggestions were considered, and adjustments were made as deemed appropriate. Consolidated Criteria for Reporting Qualitative Studies (COREQ) was used to provide a comprehensive report [27]. Finally, to ensure transparency [28], each subcategory was enhanced with citations. Each citation is in reference to the specific numbered interview. An example of the construction of the coding scheme is presented in Fig 1.

### Ethical considerations

The Research Committees of two universities approved the ethical application for Scientific and Ethical Issues (RCSEQ, no. 2117) of the University of Medicine, Health and Technology (UMIT), Hall in Tirol, Austria, and the Ethical Committee, Faculty of Natural Sciences, Department of Nursing, University of Shkodra (Prot,no. 20/2. Dt. 12/04/2018) Shkoder Albania. The study was conducted following the tenets of the Declaration of Helsinki [29] and had the approval of the head of each institution. Confidentiality of the collected data was ensured, and written informed consent was obtained from all research participants. In addition, participants were free to decline participation or withdraw at any stage of the research process. All interviews were recorded with the permission of the participants, and all the audio files were securely stored in password-protected computers.

## Results

### Demographic characteristics

The findings were extracted from the analysis of 20 in-depth individual interviews with nurses. The characteristics of the participants are presented in Table 3.

**Table 3:** Participant characteristics.

Participant	Gender	Age (y)	Education	Workplace	Work experience (years)
**1**	F	23	Bachelor	Private Nursing Home-1	1
**2**	M	32	Master	Palliative Care Center-1	10
**3**	F	44	College Diploma	Public Nursing Home-1	10
**4**	M	45	College Diploma	Public Nursing Home-1	20
**5**	F	50	College Diploma	Public Nursing Home-2	23
**6**	M	26	Bachelor	Private Nursing Home-1	2
**7**	M	25	Master	Private Nursing Home-2	2
**8**	F	33	Bachelor	Public Nursing Home-2	12
**9**	F	32	Master	Palliative Care Center-1	5
**10**	F	31	Master	Palliative Care Center-2	7
**11**	F	43	Master	Palliative Care Center-2	25
**12**	M	60	College Diploma	Public Nursing Home-2	35
**13**	F	57	College Diploma	Public Nursing Home-2	38
**14**	F	55	College Diploma	Public Nursing Home-3	18
**15**	M	30	Master	Public Nursing Home-3	8
**16**	M	25	Master	Public Nursing Home-1	1
**17**	M	26	Master	Private Nursing Home-2	2
**18**	F	25	Bachelor	Daily Elderly Care Center	2
**19**	F	25	Bachelor	Daily Elderly Care Center	1
**20**	M	24	Bachelor	Daily Elderly Care Center	1

**Fig 2:**
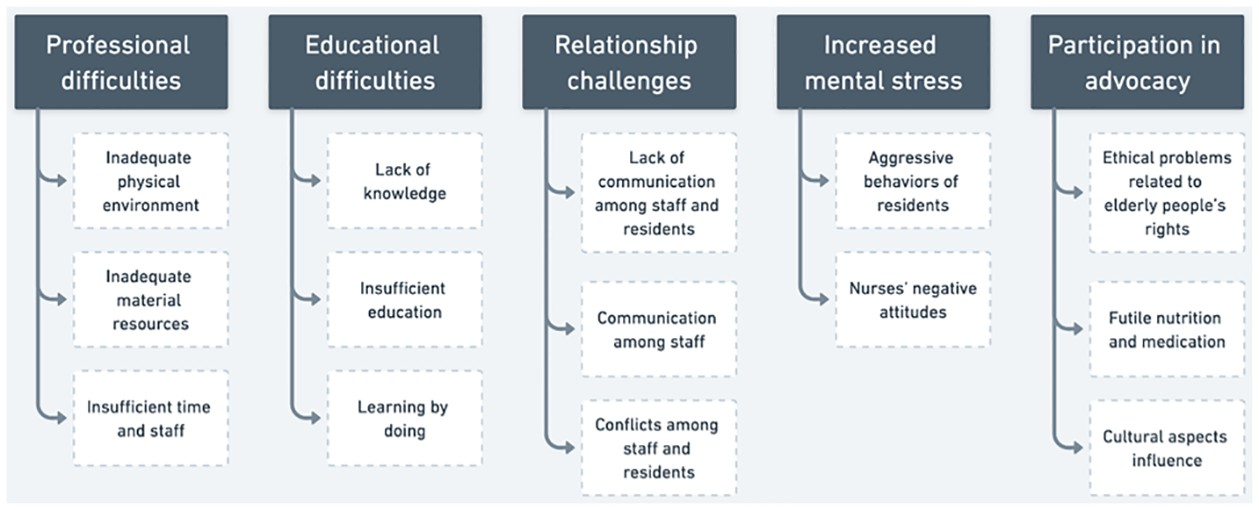
Categories and corresponding subcategories.

Five categories with fourteen corresponding subcategories were developed to capture nurses’ difficulties and challenges when caring for elderly people in different eldercare institutions in Albania (Fig 2).

### Professional difficulties

Within the realm of professional challenges, nurses highlighted three primary subcategories: inadequate physical environment, insufficient material resources, and constraints related to time and staff. All participants consistently pointed out various obstacles when caring for older adults, such as a shortage of staff, an overwhelming number of residents, time constraints, limited space, and inappropriate structural conditions.

#### Inadequate physical environment

One major issue highlighted by the nurses is the lack of suitable facilities in Albania, aside from private institutions, for providing essential care to the elderly. The environmental conditions within these institutions are not conducive for nurses to effectively carry out their responsibilities. The interviewed nurses work in nursing homes, daily elderly care centers, and palliative care centers. These institutions accommodate residents of various ages, many of whom suffer from mental illnesses or other health conditions.

In this study, certain participants shared challenges related to the physical design and layout of the ward environment, particularly in creating a familiar setting to address responsive behaviors in residents. *“They want to stay in single rooms*, *but the center doesn’t have such conditions because of the lack of funds”* (Participant 16). Participants stated that it was difficult to respect residents’ preferences and even intimacies who share rooms with other residents with dementia. *“Because the facility does not have private rooms or good conditions*, *we respect their preferences as much as possible*. *Residents stay in double rooms*. *However*, *older residents with severe conditions like paralysis or advanced dementia have quads*. *Old females remain separated from the males*, *but their intimacy is maintained only when they go to the bathroom”* (Participant 3).

#### Inadequate material resources

The participants confronted the challenge of insufficient resources, hindering their ability to provide adequate care for the elderly. Nurses felt that they were pushing the limits of their patients’ safety due to the unavailability of the necessary equipment. They were also angry about the systematic factors that did not provide them with sufficient wages and the support that they needed. *“There are even residents who drink and then get drunk*. *They are a problem*, *especially when working nights*. *Because our institution has no entertainment*, *they ask us to go out and play dominoes*. *They disturb others*, *or they even cause violence*. *This happens because we can’t do anything*, *and no one helps or supports us because of the disorganization and the lack of the necessary materials to offer a daily life to the residents“(*Participant 4).

#### Insufficient time and staff

The participants highlighted the challenge of inadequate staffing levels, with most expressing concerns about insufficient numbers of nurses and social workers per resident. *“We can’t always give care when they need it*. *We don’t have enough staff in one shift“(*Participant 8). *“There should be more staff*. *One nurse has to do almost everything*. *With only one nurse and one care worker*, *there are thirty people”* (Participant 5). Nurses reported difficulty caring for elderly people due to workload, too many residents, and lack of time and staff. *“We were warned to take care of them*. *But it’s too tricky because two people can’t monitor fifty elderly people“(*Participant 15). In addition, the lack of staff, time, and increased workload made it difficult for nurses to focus on qualitative care. Some reported feeling guilty about not being able to provide the best care for elderly people. *“The most common problem is their need for attention and their wishes to be fulfilled*. *Considering the time and workload*, *we try to fulfil their wishes somehow”*(Participant 18). *“It is a challenge to listen to the old residents*. *The staff has to provide services to them*. *We don’t have enough time to make sure we’re doing right“(*Participant 20). *“Sometimes we feel guilty*. *But the negligence in the care of the older people happens because of the lack of staff*. *Because of the lack of funds*, *the staff is insufficient”*(Participant 19).

### Educational difficulties

Under this category, three major subcategories were identified to explain educational issues: lack of knowledge, inadequate education, and learning by doing.

#### Lack of knowledge

The participants underscored a significant challenge related to the lack of knowledge in geriatric care, emphasizing that university curricula did not adequately equip them with expertise and specialization in caring for the elderly. *“Even at the university*, *there was a lack of information about caring for older adults*. *Perhaps because we were the first generation*, *this may have happened*. *The university is not consolidated*. *We need to get knowledge*, *especially for older people with dementia*, *Parkinson’s*, *and all the other diseases*. *We need to be specialized senior nurses”* (Participant 11). *“We first participated in the first training regarding caring for older adults 20 years ago*. *We need to get trained and informed more”* (Participant 14).

#### Insufficient education

Participants highlighted a significant issue related to insufficient education in geriatric care, expressing concerns about the lack of training that can impact the quality of care provided to elderly residents. One nurse emphasized this challenge, stating, *“I have not taken part in any training related to the care of the elderly*. *I have tried to find a course related to these issues*, *but the classes I have attended have not been helpful*, *considering my profession*. *However*, *we are more aware of what we should and shouldn’t do*. *Without information about such cases*, *you can even do things wrong*. *Even if we learn from our work*, *we still need proper training to fulfill our duties and protect our residents“(*Participant 9). Participants advocated for continuous education not only for staff but also for the elderly residents. One participant expressed the need for training in caring for and communicating with older adults, emphasizing that this aspect had been neglected in the past: *“I think the staff and the older people need to be trained in caring for and communicating with older adults*, *something which has never happened before”* (Participant 11). Nurses asked for training in caring for elderly adults, especially dementia care. As one nurse noted, *“We haven’t been trained on how to care for older adults or how to care for dementia*. *There is a lack of information in the college*. *It can improve the quality of care we give to the elderly and help us to be more confident and professional if we have knowledge and training about elderly care*, *especially dementia care”* (Participant 1). The participants underscored the need for professional education to provide specialized care to elderly people, particularly those with dementia at different stages and symptoms. Despite attending some training sessions, they expressed a desire for more comprehensive education in caring for older adults. One nurse noted, *“We’ve attended training on diagnosing the patient*, *but it wasn’t explicitly related to the elderly”* (Participant 15). “*We recently attended a training on the standards we should meet*, *organized by two psychology professors*. *But we need more training in caring for older adults*. *The problem is that our institution does not offer proper care for the elderly*, *and we*, *the nurses*, *are not even specialized in caring for the elderly”* (Participant 13).

#### Learning by doing

A prevalent theme among participants was the concept of learning by doing, indicating that their knowledge and skills in caring for older adults were primarily acquired through practical experience rather than formal education. Participants acknowledged the valuable contributions of previous staff, emphasizing their higher education and multilingual abilities. One participant stated, *“We also learned a lot from the previous staff*. *They had higher education and could speak many languages”* (Participant 12). Nurses learned to solve unforeseen problems while caring for older adults without guidance or resources or only through their family education: *“Human resources were too limited in terms of staff and professors*. *We learned by working*, *although we did not have enough information*. *I think we got something done”* (Participant 2). *“I don’t have any update on elderly care even though I have been working here for almost thirty years*. *We learn by working and practicing it daily”* (Participant 4). *“Rather than being trained in an institution*, *we have been working based on our family training”* (Participant 2).

### Relationship challenges

Many nurses cited staff-resident-family relationships as barriers to appropriate care for older adults. They emphasized the lack of communication and conflict between and among staff, residents, and families.

#### Lack of communication among staff and residents

Numerous participants highlighted the communication challenges they face, particularly when interacting with elderly individuals diagnosed with dementia. While nurses acknowledged the importance of patience in these situations, time constraints often hindered their ability to engage in meaningful communication. The need to prioritize residents’ physical needs was emphasized by one participant, who stated,*“I will listen to them*, *but if I am in charge of something else*, *I will tell them to wait if it is not urgent*. *Sometimes*, *they’re reasonable*. *Other times*, *they act like they don’t understand what I’m saying*. *We have to prioritize their physical needs”* (Participant 9). The difficulties in communication were further compounded by the diverse range of residents with varying ages and health conditions. Instances of alcoholism, previous incarceration, and both physical and mental disabilities among residents contributed to communication problems. The nursing staff, despite facing these challenges, made efforts to adapt to the residents’ diverse needs. However, the complexity of their job increased when residents encountered problems among themselves, requiring intervention and separation. As described by one participant, *“Some people here are alcoholics or have been in prison*, *as such we may have communication problems with them*. *We also have people with physical and mental disabilities*. *Even though it’s difficult*, *we try to adapt ourselves to them*. *In general*, *they’re not aggressive*, *but there are cases in which they have problems with each other*. *Sometimes*, *we have to intervene and separate them from each other”* (Participant 8).

This theme underscores the intricate nature of communication challenges within eldercare institutions, emphasizing the diverse backgrounds and conditions of the residents that contribute to the complexity of interactions for nursing staff.

#### Communication among staff

Regarding communication among staff members, most participants expressed overall positive communication within the team. However, some nurses acknowledged minor disagreements arising from different positions and cultural backgrounds. One nurse highlighted this aspect, stating, *“In some ways*, *we face problems communicating with each other because we have different cultures and ways of caring for patients*. *I take care of them as a nurse*. *Another staff member helps as a caretaker”* (Participant 17).

Meanwhile, communication between nurses and doctors was generally perceived as effective and correct. Nurses reported rarely experiencing conflicts with doctors, emphasizing that doctors typically make decisions and nurses follow their instructions. As described by one participant: *“We never happen to conflict with the doctors*, *and we are better at communicating with them”* (Participant 7). The collaborative dynamic between nurses and doctors contributes to the smooth functioning of the team.

#### Conflicts among staff and relatives

Numerous participants highlighted challenges in communication and misunderstandings with the families of residents, particularly those with dementia. The participants expressed frustration by stating: *“We sometimes have problems communicating with the families because they do not accept the situation of their relatives“(*Participant 10). Conflicts with family members were reported, often stemming from dilemmas faced by relatives regarding the care of their loved ones. One respondent emphasized the complexities involved: *“Sometimes the relatives are in a dilemma whether to do the right thing or not to bring their father or mother*, *so they often need us to listen or confirm when they’re right*. *There are cases in which family members impose things on the patients*. *For example*, *they force them to eat more than they want*, *which is a source of conflict for us”*(Participant 2).

### Increased mental stress

Nurses in this study mentioned their psychological stress while caring for elderly residents with various conditions. They emphasized the residents’ aggressive behavior and the nurses’ negative attitudes.

#### Aggressive behaviors of residents

Participants recounted distressing experiences involving aggressive behaviors exhibited by residents and their families. One participant highlighted the challenge, stating, *“There are cases when family members don’t cooperate with us and insult us*. *This makes our job even more difficult”* (Participant 18). Despite understanding that aggressive behavior in elderly residents is often a consequence of dementia, nurses reported feeling hurt and demoralized. Instances of verbal insults, physical aggression, and biting were mentioned, creating emotional challenges for the nursing staff: *“We care for elders and treat them like family members*. *Sometimes they insult us verbally and become aggressive*. *They hit us or bite us*, *and when they do that*, *we feel bad and insulted”* (Participant 15). While acknowledging the difficulties, nurses emphasized the importance of effective communication in managing such situations. One nurse stressed: *“I think it’s more important than anything else to be able to communicate*. *Every older resident has their character*. *Some seniors are grateful to us for our services*.

*On the other hand*, *some of them may throw food at our uniform if we don’t do what they want*. *Sometimes*, *there are even insults*. *But let’s not consider their abuses“(*Participant 12). *“Yes*, *sometimes we have aggressive residents*. *However*, *we do our best to calm them down through conversation*. *We try not to give them tranquilizers*, *but sometimes we have to use them and feel guilty about it*…*there is no other way”* (Participant 6).

#### Nurses’ negative attitudes

Most participants highlighted concerns about the negative attitudes exhibited by some nursing staff, emphasizing a lack of a sense of mission and emotional engagement. This was seen as a hindrance to holistic care, reducing care to mechanical and physical tasks. One participant stated: *“In my opinion*, *the attitude of some of the nurses was wrong*. *They do not have a sense of mission or a sense of professional duty; they have no emotions”* (Participant 1). *“I have been working here for an extended period*. *Sometimes*, *it looks like I’m acting like a robot*… *When we care for residents with dementia*, *we have to talk to them more*, *listen to them*, *and give them some stimulation*. *Still*, *sometimes I feel pressured by the resident*, *relatives*, *or other staff*. *I change diapers*, *take residents to physiotherapy*, *and feed them lunch or dinner; lots of stress*, *that’s all*. *I’m sorry*, *but many residents need me to help them physically“(*Participant 13).

### Participation in advocacy

The participants indicated that they are often confronted with ethical issues when they advocate for their elderly residents. These are listed in three subcategories: ethical problems related to elder rights, futile nutrition and medications, and cultural influences.

#### Ethical problems related to elderly people’s rights

Nurses highlighted ethical dilemmas related to older people’s rights, particularly in institutionalization cases where informed consent may be lacking. The challenge lies in the fact that many elderly individuals are institutionalized without being adequately informed or asked for their consent. Nurses expressed the difficulty of defending the rights of older people in such situations. According to one participant: *“Most of them are here at the request of their family members*. *The family members often give them the wrong information by telling them that our center is a hospital*. *When we tell them the truth*, *they understand the reality as the days pass”* (Participant 7). *“Family members usually decide to bring their parents to our center*. *They may be abroad or busy with their work*. *But we can’t keep them against their will*, *so we also try to get their consent”* (Participant 6). *“The official document is part of every palliative care report*. *But we never use it because the resident is not informed*, *and we cannot do anything wrong*. *The older adult must be well informed about what he has and what we serve him*. *It’s the patient’s right to sign in*, *and he must accept everything with full awareness*. *We haven’t used an official document very often*. *Most colleagues do not know much about it“(*Participant 11).

#### Futile nutrition and medication

Feeding and futile therapies were other challenges expressed by the participants. Nurses said they often have to feed elderly people even when they do not want food. In some cases, family members insisted on providing food to unconscious patients, believing it would contribute to their well-being. *“This happens because the patient is unconscious at the family member’s request in the last phase*. *There have been cases in the last days of life where patients have refused to eat*. *However*, *we must feed them*, *even though we cannot give them basic food*, *but bland food for better treatment”*(Participant 17). *“The relatives insist on giving the patient futile therapy until the last moment of his life*, *even though they know we don’t give curative therapy*. *The doctor must tell them what’s best*. *However*, *the relatives are supposed to decide*. *I always try to convince the families not to give the patient such things*. *They are worthless and only harm the patient*. *But if they still insist on it*, *I try to give the patient the minimum of the doctor’s prescribed treatment”* (Participant 8).

In certain instances, nurses also argued that elderly residents receive futile therapy that may harm rather than benefit them. However, the decision lies with the family: *“Some residents have dementia*, *crises*, *or emotional problems*. *If we consult the doctor*, *we can treat with or without the patient’s consent*. *This makes me feel horrible because I do nothing to advocate for my old resident”* (Participant 19). *“Older adult residents who have dementia are refusers of treatment*. *When this happens*, *we try to find ways to make it possible for them to receive the medication without consent*. *For example*, *we could give them the medicine in their food*. *I know this doesn’t seem right and has nothing to do with my role as advocator*, *but I am forced to do so*” (Participant 16).

#### Cultural aspects influence

Nurses participating in the study highlighted significant challenges arising from cultural aspects that impact their ability to advocate for elderly residents in Albanian eldercare institutions. The participants expressed a sense of powerlessness in advocating for residents with no family to request visits to the facility. Moreover, cultural norms and taboos restrict the disclosure of significant health problems and diagnoses to elderly individuals, emphasizing the influence of societal beliefs on communication and decision-making in healthcare settings. *“Nobody asks them if they want to come here*. *The government brings them here*, *and we have to accept them in our institution without any advocacy regarding information or other things”* (Participant 13). *“Based on the information we get*, *we try to be compassionate and polite*. *We are somehow restricted from discussing the pathology with the patient because of the taboos and mentality of our society*. *Patients often accept the information given to them by family members and do not ask many questions*. *This is a part of the culture in Albania*. *So*, *we give patients the news their relatives want them to know*. *We do nothing to protect the older adult residents here*, *which makes me feel terrible”* (Participant 3). *“Because the familiars insist on not telling them the truth*, *most of our residents do not have this information about their diagnosis*. *They ask us not to tell patients the truth*. *However*, *this phenomenon of not telling the truth about the diagnosis to the patient is part of the Albanian culture*. *It is considered a problem for us”* (Participant 1).

## Discussion

This qualitative study sheds light on the multifaceted challenges faced by nurses in Albanian eldercare institutions, categorizing their experiences into five key areas. Following previous literature, these results showed that the work environment impacts nurses’ work and attitudes toward caring for older adults [30, 31]. Likewise, in their studies, Rush et al. (2017), Adibelli & Kılıç (2013), and Kong et al. (2022) found that the physical work environment influenced the work of the nurse and their attitude toward caring for elderly people and their health [30, 31]. According to this study’s findings, Albanian eldercare institution leaders, policymakers, and other stakeholders need to consider the importance of the physical environment, redesign the institutional setting to be more appropriate, and make the institution more home-like for elderly people. In an attempt to provide high-quality care, it is necessary to have enough nurses [14, 31]. The participants in this study were overloaded with work and did not have enough time to care for the elderly because of inadequate standards and time to provide proper care. They reported feeling guilty for not providing the best care due to insufficient time and staff.

Similarly, Kang & Hur (2021) found that the shortage of nurses prevents communication and care for older persons, especially those with dementia [17]. The results of this study show that Albanian eldercare institutions need adequate staffing standards and financial support to implement appropriate care for elderly residents successfully. This study revealed that the participants had inadequate education and training in caring for the elderly, especially those with different mental and physical conditions. Most of the participants highlighted problems related to a lack of knowledge and skills in eldercare, especially in caring for older people with dementia. These results are reinforced by other authors’ findings, who emphasized the importance of staff and elderly education in elderly care in their studies [17, 30, 32–34]. Thus, to provide the best care for older people, the leaders of Albanian eldercare institutions and universities should provide continuous training on care for the elderly.

Adequate care requires excellent and healthy communication, mutual trust and understanding, and good teamwork and relationships [14, 35, 36]. Similarly to previous studies, this study found that nurses experienced relationship and communication difficulties with residents, especially those with dementia and their families [14, 17, 30, 32, 33]. These difficulties are mainly related to the Albanian culture, where families care for elderly people. Likewise, Adibelli & Kılıç (2013) and Nasrabadi et al. (2021) describe these difficulties as primarily related to cultural problems [30, 37]. In addition, the nurse participants in this study reported issues of communication and relationships within the team. These findings support previous reports on the importance of communication, relationships, and collaboration while caring for elderly people [14, 38]. Eldercare institution leaders need to recognize the importance of their role in team relationships and provide education and support to improve communication and relationships between staff, residents, and their families.

Another result showed that most of the nurses included in this study suffered from increased psychological stress when caring for elderly residents, especially those with various mental health problems, such as dementia. Emotional distress and stress were experiences of nurses whom the aggressive behavior of some residents with dementia had hurt. Adding to previous findings, participants in this study reported hurtful experiences while caring for some elderly people [14, 17, 35]. These experiences led to negative attitudes among nurses, who consistently said they were apathetic and mechanical in treating residents with dementia. They also had difficulty treating and respecting them as human beings. Accordingly, nurses who experienced aggressive behavior from elderly residents felt anxiety, frustration, and discomfort while caring for them, according to Kang & Hur’s (2021) meta-synthesis [17]. Consistent with Kang & Hur’s (2021) findings, participants in this study reported difficulty communicating with residents in the face of aggression. Therefore, measures such as using safety alarm devices and counseling programs to support nurses’ psychological and physical health should be taken to protect nurses facing emotional stress and provide qualitative care [14, 17].

Another significant challenge described by participants was advocacy related to ethical issues in caring for older adults in long-term care facilities. They found it challenging to advocate for elderly residents regarding self-determination, truth-telling, and trust. Along the same lines, Arcadi & Ventimiglia (2017) and Luca et al. (2021) emphasized the need for nurses to develop a trusting relationship [16, 39]. Participants in this study believed that advocacy skills are needed when ethical issues arise, such as when the needs of the elderly are not met or considered, as also mentioned by Josse-Eklund et al. (2014), when excessive or unnecessary therapy may occur against the expressed will of the patient, or when patients with dementia are unable to express their will regarding therapeutic decisions made by family members or physicians [40]. In such situations, participants emphasized that they cannot protect the rights of elderly residents, most of whom are placed in institutions against their will and without their consent. Family members make decisions, although older people have the right to information, choice, and approval following relevant regulations and laws [9]. Despite the various conflicts nurses can encounter with colleagues or families, the importance of nurses’ ability to guarantee and protect the rights and wishes of older people in the centers where they work has been described and highlighted in earlier studies [16]. Managers of facilities for eldercare should consider this fact, and more support should be provided. In addition, participants in this study believed that cultural aspects such as social relationships influence the views of the patient, family, and nurses on a clinical situation and cause different difficulties for nurses in caring for the elderly. Indeed, Vries et al. (2019) support these findings, arguing that cultural aspects such as ethnicity, religiosity, and spirituality, as well as the level of health literacy, greatly influence people’s decisions when making plans that consist not only of advanced directives for resuscitation but also wishes at the end of their lives [7, 9, 41]. In the present study, despite the different organizational contexts of the eight eldercare institutions, the participants described similar challenges in caring for elderly residents. The results of this study indicate that Albanian eldercare institutions / long-term care facilities need to develop a supportive organizational system and provide support for the successful implementation of elderly care. Furthermore, the Albanian government must offer more financial and legitimate aid for eldercare institutions.

### Limitations and strengths

This study comes with some limitations commonly found in qualitative studies. Firstly, it relies predominantly on the perspectives of nurses, potentially limiting the exploration of broader frameworks and contextual conditions. Secondly, as a qualitative study, this study is limited in scope and relies on the subjective interpretations and decisions of the involved researchers. Additionally, the study is confined to eight eldercare institutions in three regions of Albania, which may restrict the generalizability of the findings to other contexts or countries.

Nevertheless, this is one of the few studies conducted in Albania regarding nursing staff challenges and difficulties in eldercare facilities. Thus, the current study creates an opportunity for further extensive national research into nurses’ knowledge, perception, attitude, and other relevant areas that may affect older people and their care in Albania. Future studies should consider the experience of all healthcare professionals involved in providing care to older people in all Albanian eldercare institutions.

## Conclusion

In this study, a significant number of nurses highlighted substantial challenges in providing care to elderly patients. The key obstacles identified encompassed the absence of specialized long-term care infrastructure, insufficient social support and funding, inadequate knowledge and training in geriatric care, and the absence of clear policy guidelines for the care of older individuals. The findings underscore the urgent need for enhanced legal and financial support at the national level and economic and educational support at the facility level to enhance staffing conditions and the physical environment in long-term care facilities, thereby improving the overall care for older individuals. Establishing suitable long-term care environments across all regions, along with comprehensive guidelines, enhanced nurses’ knowledge, improved working conditions, and ongoing training are imperative. Initiatives should include reinforcing geriatric nursing programs in Albanian universities to adequately equip professionals in addressing the healthcare requirements of the elderly. Long-term care facilities should implement geriatric education at the facility level for staff and families, focusing on knowledge enrichment, improved communication skills, and ethical problem-solving practices. Leaders of these facilities play a pivotal role in recognizing and addressing the challenges faced by nurses, emphasizing their crucial contribution to ensuring quality care for older individuals.

## Supporting information

S1 File (DOC)
